# Evidence-based sizing of non-inferiority trials using decision models

**DOI:** 10.1186/s12874-018-0643-2

**Published:** 2019-01-07

**Authors:** Iris Lansdorp-Vogelaar, Reshma Jagsi, Jinani Jayasekera, Natasha K. Stout, Sandra A. Mitchell, Eric J. Feuer

**Affiliations:** 1 Department of Public Health, Erasmus MC University Medical Center Rotterdam, Rotterdam, the Netherlands; 2 University of Michigan, Ann Arbor, MI USA; 3 Georgetown University Medical Center, Washington, DC USA; 4 Department of Population Medicine, Harvard Medical School and Harvard Pilgrim Health Care Institute, Boston, MA USA; 5 Healthcare Delivery Research Program, Division of Cancer Control and Population Sciences, National Cancer Institute, Bethesda, MD USA; 6 Statistical Research and Applications Branch, Surveillance Research Program, Division of Cancer Control and Population Sciences, National Cancer Institute, 9609 Medical Center Drive, Room 4E534, Bethesda, MD 20892-9765 USA

**Keywords:** Non-inferiority trial, Sample size, Power calculation, Quality-adjusted life years, Non-inferiority margin

## Abstract

**Background:**

There are significant challenges to the successful conduct of non-inferiority trials because they require large numbers to demonstrate that an alternative intervention is “not too much worse” than the standard. In this paper, we present a novel strategy for designing non-inferiority trials using an approach for determining the appropriate non-inferiority margin (δ), which explicitly balances the benefits of interventions in the two arms of the study (e.g. lower recurrence rate or better survival) with the burden of interventions (e.g. toxicity, pain), and early and late-term morbidity.

**Methods:**

We use a decision analytic approach to simulate a trial using a fixed value for the trial outcome of interest (e.g. cancer incidence or recurrence) under the standard intervention (p_S_) and systematically varying the incidence of the outcome in the alternative intervention (p_A_). The non-inferiority margin, p_A_ – p_S_ = δ, is reached when the lower event rate of the standard therapy counterbalances the higher event rate but improved morbidity burden of the alternative. We consider the appropriate non-inferiority margin as the tipping point at which the quality-adjusted life-years saved in the two arms are equal.

**Results:**

Using the European Polyp Surveillance non-inferiority trial as an example, our decision analytic approach suggests an appropriate non-inferiority margin, defined here as the difference between the two study arms in the 10-year risk of being diagnosed with colorectal cancer, of 0.42% rather than the 0.50% used to design the trial. The size of the non-inferiority margin was smaller for higher assumed burden of colonoscopies.

**Conclusions:**

The example demonstrates that applying our proposed method appears feasible in real-world settings and offers the benefits of more explicit and rigorous quantification of the various considerations relevant for determining a non-inferiority margin and associated trial sample size.

## Background

Traditionally, trials investigating de-escalation of interventions have employed a non-inferiority design, requiring large numbers to convincingly demonstrate that an alternative treatment is “not too much worse” than a standard treatment with known benefit. This definition of benefit conventionally focuses on disease control and/or prevention outcomes alone, without explicit quantification or consideration of the differential impact of different regimens on toxicity, burden, and early and late-term morbidity.

Determination of the non-inferiority margin is the most critical step in non-inferiority testing, as it represents the point of equipoise where the benefits of the standard therapy compared to the lesser alternative are outweighed by its risks or perhaps its additional costs [1].

While there are several guidelines available to aid researchers in the development of non-inferiority margins in these trial designs [2–9], a recent systematic review of non-inferiority trials showed that the majority of previously published trials have either provided ambiguous or limited information to justify their choice of margin [10]. Lack of robust justifications for setting non-inferiority margins in standard practice could lead to inconsistency in recommendations and guidelines based on non-inferiority trials. More importantly, current guidelines do not provide strict standards for considering risks, morbidity and costs in the determination of non-inferiority margins, necessitating large clinical trials to evaluate these sorts of questions.

In this paper, we introduce a general decision analytic framework that uses an explicit net-benefit approach for determination of non-inferiority margins for optimal design of non-inferiority trials in oncology as well as other disease settings. In this context, quality-adjusted life years (QALYs) can be used as a single integrated measure of health outcomes that represent both the quality and quantity of life lived [11]. If QALY loss of a current intervention compared to an alternative are substantial, a larger cancer-control benefit would be necessary to justify its continued use.

In this paper, we introduce a novel methodology based on decision-modeling for setting the non-inferiority margin of trials investigating de-escalation of interventions. Examples of such trials are those that investigate: 1) omitting (parts of) the treatment regimen, 2) lower drug-dose regimens, 3) alternative drugs with less side effects and 4) lower frequency of follow-up exams, In the next section, we briefly describe the general concepts behind the methodology before illustrating its applicability.

## Methods

### Non-inferiority trials and the non-inferiority margin

The traditional approach to non-inferiority trials in oncology tests whether a new experimental treatment is not meaningfully worse than an existing treatment in terms of a disease outcome (e.g. cancer recurrence rate) [12]. The concept of “meaningfully worse” is formalized in the definition of a value called the non-inferiority margin, or more generally the equivalence margin, denoted by δ. The non-inferiority margin defines the maximum clinically acceptable difference that one is willing to accept in return for the lower burden, morbidity and/or costs of the new therapy [1]. Non-inferiority trials have a null hypothesis that the alternative treatment is inferior to the standard treatment by at least the pre-specified non-inferiority margin (Ho: p_A_ – p_S_ > δ, where p_S_ and p_A_ are event rates of the outcome of interest for standard and alternative interventions, respectively). The alternative hypothesis is that the alternative treatment is not inferior to the standard treatment (i.e., is less than the non-inferiority margin (Ha: p_A_ – p_S_ < δ)).

Several methods have been proposed for setting the non-inferiority margin. Two methods, the 95–95 method and the synthesis method, are prescribed by the FDA in the evaluation of new interventions to ensure that the non-inferiority margin does not overlap the event rate in a control population [9]. Neither of these methods account for the difference in morbidity outcomes between the interventions, so even if the new intervention is better than the placebo in terms of mortality or event rates, the margin does not guarantee that the new intervention is truly non-inferior to the current intervention. Thus, while these methods are useful to ensure that the new intervention is superior to a control, it does not obviate the need to still quantify the non-inferiority margin in terms of the harms and benefits of the standard and new intervention. The methods can therefore be considered additional constraints.

The Delphic method does take morbidity into account, as it asks physicians or patients to subjectively assess how much benefit they might forgo to avoid the potential incremental harms of the standard therapy [12]. However, it may not do so in a reproducible, systematic way, especially with respect to the long-term implications of the interventions being compared. Setting a framework for an evidence-based quantification of the non-inferiority margin is therefore the focus of this paper.

### Proposed decision analytic approach

Our proposed framework utilizes a decision analytic approach to simulate a trial using a fixed value for cancer incidence or recurrence rates under the standard intervention (p_S_) and systematically varying cancer incidence\recurrence under the alternative intervention (p_A_). The non-inferiority margin, p_A_ – p_S_ = δ, is reached when the lower cancer incidence or recurrence rate of the standard therapy is counterbalanced by the higher disease rate but improved morbidity burden of the alternative. This is quantified in a decision model as the level of incidence/recurrence (p_A_) at which the QALYs in the two arms are equal. Therefore, true non-inferiority, as operationalized in this paper, is established when the potential loss in life-years due to lower efficacy in the alternative intervention is offset by an increase in quality of life from lower burden and/or side effects relative to the standard intervention. While some trials have used outcome measures of net benefit, this paper advances the literature by using decision analytic models and measures of net benefit to explicitly quantify the non-inferiority margin, and, by extension, formally size the trial.

**Fig. 1:**
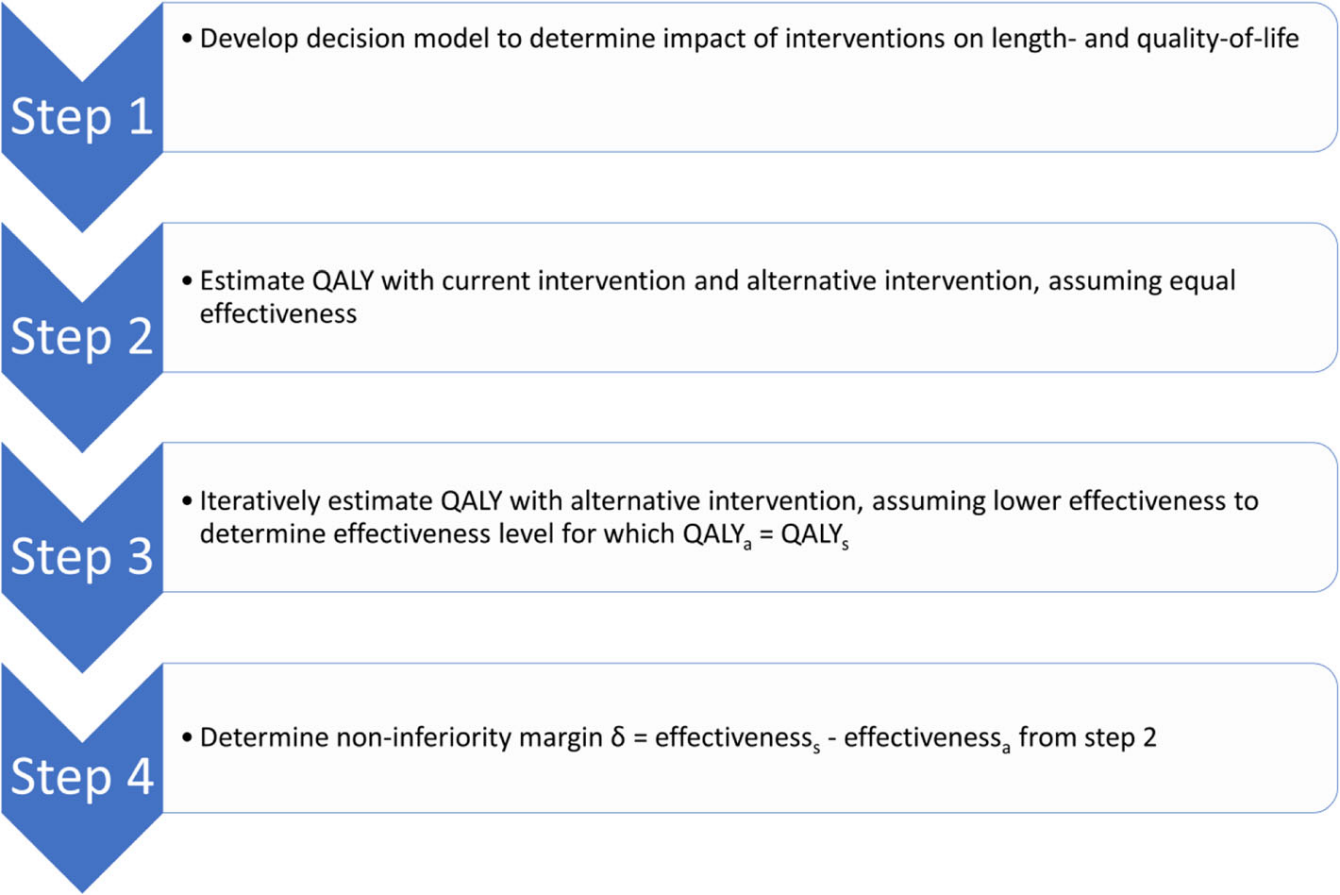
Overview of four steps of proposed methodology to determine evidence-based non-inferiority margin in non-inferiority trials. “s” represents the standard intervention and “a” represents the alternative scaled back intervention

Our proposed framework consists of four steps (Fig. 1), which are illustrated with an example in the next section. In Step 1, a decision model is formulated as a framework for quantifying the lifelong impacts of outcomes by integrating the probabilities of specific outcomes and their sequalae with their disutilities. An existing decision model can be used, or one can be developed de novo. Disutilities can be obtained from previous studies or literature. A model can be simple, including only the relevant types of adverse events and their associated disutilities post cancer diagnosis over the remaining life course.

In Step 2 we use the decision model to estimate how the alternative intervention would improve the quality of life of the patient, if it would have the same effectiveness as the current intervention.

In Step 3, the model is used to estimate quality of life under the assumption that the alternative intervention is slightly less effective than the standard intervention, and therefore may result in either more deaths or earlier death with an associated decrease in length of life. A less effective alternative intervention may also result in additional incident or recurrent cases of the disease and thus a loss in quality of life on top of the loss in average length of life. By iteratively evaluating the effectiveness of the alternative intervention, we can find the point at which the QALYs lost from the lower effectiveness is equal to the QALYs gained from the lower burden and/or side effect of the alternative intervention. Finally, in Step 4, the difference in effectiveness between the current intervention and the derived effectiveness level for the alternative intervention can then be used as the non-inferiority margin for evidence-based sizing of non-inferiority trials.

### Alternative measures for non-inferiority

Using disutilities from literature for determining equal QALYs implies that a current intervention may be replaced by an alternative when it is non-inferior for those whose utility weights do not diverge dramatically from the average member of the population. A more conservative approach would be to extend the trial applicability to a larger fraction of the population, which would require down weighting the disutility associated with the toxicity or burden of the intervention relative to the average population, leading to a smaller non-inferiority margin and therefore a larger sample size. Analyses along these lines are possible and would be similar to the one described above, but instead of using average disutilities, they might consider utility weights derived from a different cut-point of the population distribution (e.g., the 25th percentile).

Alternative interventions or omission of an intervention may not just be less harmful but may also be less expensive for the target population. As such, a non-inferiority margin could also be designed if lower costs could make up for a certain reduction in effectiveness. The decision analytic method is largely the same as above, except that the margin is determined based on cost-effectiveness (costs per QALY) rather than QALY. The non-inferiority margin is established based on the level of effectiveness such that the reduction in costs no longer compensates enough for the reduction in QALYs given a willingness-to-pay threshold (e.g., the ratio is at or above a threshold).

### Uncertainty analysis

Some parameters assumed to be known in the model, in fact are not known with certainty. To account for this uncertainty, one can repeat the proposed approach for different parameter values. This way, insights are obtained on the sensitivity of the power calculation to different model assumptions. To ensure sufficient power, one could consider using a sample size in the upper ranges of the uncertainty analysis.

## Results

We chose the European Polyp Surveillance (EPoS) study [13] as an example of a non-inferiority trial to illustrate how this decision-analytic approach can help inform the non-inferiority margin and design of non-inferiority trials. We used the MISCAN-Colon microsimulation model as the decision analytic model to estimate QALYs and costs for this example [14]. We first briefly describe the trial and the model before presenting results with respect to applying the framework to establishing an evidence-based non-inferiority margin.

### EPoS trial

The EPoS study consists of two ongoing randomized controlled trials and a planned cohort follow-up study. A detailed description of the design of the trial was recently published [13]. In this example, we focus on the EPoS I randomized controlled trial. In brief, in EPoS I, low-risk adenoma patients (i.e., patients with 1–2 small tubular adenomas without high-grade dysplasia or villousness) were randomized to receive surveillance colonoscopy at both years 5 and 10 (standard intervention) vs. surveillance colonoscopy at 10 years only (suggested alternative scaled-back intervention).

The study was powered as a non-inferiority trial, since the investigators wanted to determine if the 10-year colorectal cancer (CRC) incidence for the scaled-back intervention fell within a specified margin of that previously observed for the standard intervention. The expected 10-year CRC incidence under the standard intervention is approximately 1%. For the power calculation, it was felt that a 10-year CRC incidence rate of up to 1.5% could be tolerated to gain the advantages of having surveillance colonoscopies half as frequently. Thus, a non-inferiority margin of 0.5% was used. Based on 90% power and a one-sided alpha of 0.05, it was estimated that a total of 13,766 individuals needed to be included in EPoS I.

### MISCAN-Colon

MISCAN-Colon is a well-established microsimulation model for CRC developed at the Department of Public Health of the Erasmus University Medical Center (Rotterdam, the Netherlands) [15, 16]. It is one of the models participating in the National Cancer Institute’s Cancer Intervention and Surveillance Modeling Network (CISNET) [17–19]. The model’s structure, underlying assumptions, and calibration are described in previous publications [15, 20]. Briefly, the model simulates the life histories of individuals from birth to death. CRC arises in the population according to the adenoma-carcinoma sequence. Screening and surveillance may alter these life histories through possible removal of adenomas and detection of cancers. In this way CRC mortality can be reduced. The life years gained by screening are calculated as the difference in model-predicted life years lived in the population with and without CRC screening.

We used MISCAN-Colon to simulate the EPoS I study population of individuals diagnosed with low-risk adenomas and undergoing subsequent surveillance. We simulated two colonoscopy surveillance strategies, one with surveillance every 5 years, and one with surveillance every 10 years. Surveillance was assumed to continue until age 75. We followed individuals for their lifetimes. The model was used to predict lifetime QALYs and costs of the standard and alternative surveillance interventions. Assumptions for natural history and costs were based on previous work [21].

### Disutilities from surveillance colonoscopy and CRC

The potential gain in QALYs from the less intensive surveillance interval compared to the more intensive interval stems from the reduction in colonoscopies required, as colonoscopies are associated with patient burden and complications. For this analysis, disutilities were chosen by assumption. We assumed a population-average disutility of 3.1 days for every colonoscopy performed to account for 3 weeks of anxiety prior to colonoscopy at a disutility of 0.1, and 2 days of preparation and procedure at a disutility of 0.5. In addition, we modeled age-specific risks for gastrointestinal and cardiovascular complications of colonoscopy. The overall risk associated with colonoscopies with polypectomy increased exponentially with age: from 2 complications per 1000 colonoscopies at age 40 to 38 per 1000 at age 85 [15]. Colonoscopies without polypectomy were not associated with a risk for complications. We assumed a utility loss of two weeks of life per complication from colonoscopy. We assumed that one out of every 30,000 colonoscopies involving polypectomy resulted in death.

On the other hand, treatment for CRC is also associated with a loss in quality of life, and higher rates of recurrence may therefore have a negative impact on QALYs. Disutilities for life-years with CRC were therefore also incorporated, based on findings by Ness et al [22].

### Determining the non-inferiority margin

#### Estimate QALYs with less intensive surveillance assuming equal effectiveness

Assuming a 1.0% risk of CRC incidence with the standard intervention of 5-yearly surveillance, the model predicted 22,424 life-years per 1000 50-year old adenoma patients. Per 1000 adenoma patients, 26.3 QALYs would be lost due to surveillance colonoscopies and another 0.3 QALYs due to complications of surveillance (Table 1). In addition, 54.1 QALYs per 1000 would be lost due to diagnosis and treatment of CRC. Together, this resulted in total of 22,343 QALYs per 1000 adenoma patients (Table 1).

**Table 1:** Comparison of QALY outcomes across various scenarios of surveillance for adenoma patients and CRC risk

Strategy	10y cumulative incidence	Life-years (per 1000 pt)	QALYs lost due to surveillance (per 1000 pt)	QALYs lost due to complications (per 1000 pt)	QALYs lost due to treatment (per 1000 pt)	QALYs (per 1000 pt)	Difference in QALYs (per 1000 pt)
5-yearly surveillance	1.0%	22,424	26.3	0.3	54.1	22,343.2	Reference Strategy
10-yearly surveillance	1.0%	22,424	18.0	0.2	54.1	22,351.5	8.3
10-yearly surveillance	1.5%	22,412	18.1	0.2	55.8	22,338.1	−5.1
10-yearly surveillance	1.3%	22,419	18.0	0.2	49.8^a^	22,351.0	8.1
10-yearly surveillance	1.42%	22,415	18.1	0.2	53.5^a^	22,343.1	0.0

10y: 10-year; QALYs: Quality-adjusted life-years; pt.: patients

^a^ QALY lost to treatment are not necessarily higher with higher CRC incidence, because they also depend on the stage distribution of CRC cases and time spent in each CRC state. Higher CRC incidence may lead to fewer life-years with CRC treatment because of higher mortality from CRC

Under an initial assumption of equal effectiveness (a 1.0% risk of CRC incidence) for both 10-yearly and 5-yearly surveillance, life-years with the alternative surveillance schedule of 10-yearly surveillance were the same as with 5-yearly surveillance, i.e., 22,424 years per 1000 adenoma patients. However, because of a reduction in colonoscopies performed, the QALYs lost to surveillance are lower (i.e., 18.1 and 0.2 for colonoscopies and complications, respectively). Consequently, QALYs for the alternative intervention were higher at 22,352 years. This amounts to an improvement in QALYs of 8.3 per 1000 adenoma patients compared to the standard intervention.

#### Iteratively determine level of effectiveness for equal QALYs

**Fig. 2:**
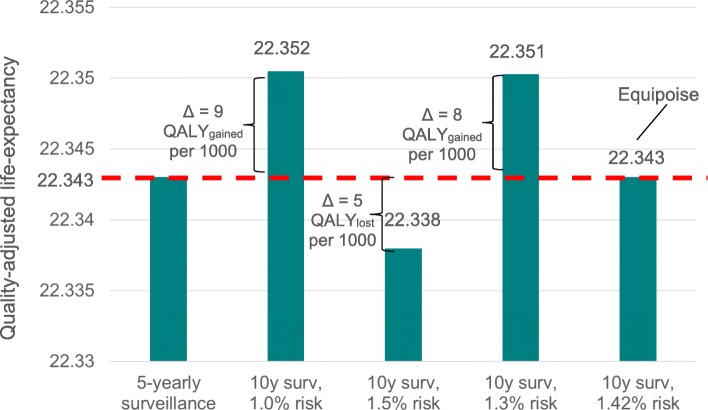
Quality-adjusted life-expectancy in low-risk adenoma patients for the current intervention and the alternative intervention at different levels of effectiveness. Current intervention is 5-yearly surveillance; Alternative intervention is 10-yearly surveillance. Different levels of effectiveness represent difference levels of CRC risk after the alternative intervention

For a 1.5% risk of CRC incidence at 10 years under 10-yearly surveillance, which was used to design EPoS I, QALYs would be 22,338 years per 1000 adenoma patients. This is slightly lower than the QALYs from the current intervention (Fig. 2). If 10-yearly surveillance would lead to a 10-year risk of CRC incidence of 1.3%, total QALYs would be 22,351 years, for a gain in QALYs of 7.8 years per 1000 adenoma patients (Table 1). Through iterative running of the MISCAN-Colon model for different levels of 10-year risks of CRC incidence in adenoma patients with 10-yearly surveillance, we found that at a risk of CRC incidence of 1.42%, QALYs from 5-yearly surveillance and 10-yearly surveillance would be equal (Table 2).

**Table 2:** Non-inferiority margins and sample size requirements for 5-yearly surveillance (vs. 10-yearly surveillance) of low-risk adenoma patients

Strategy	10y cumulative incidence	Life-years (per 1000 pt)	QALYs lost to surveillance (per 1000 pt)	QALYs lost to surveillance complications (per 1000 pt)	QALYs lost to CRC treatment (per 1000 pt)	QALYs (per 1000 pt)	Required sample size
Non-inferiority based on effectiveness and average disutilities (base case) – 3.1 days lost per colonoscopy							
5-yearly surveillance	1.0%	22,424	26.3	0.3	54.1	22,343	19,234
10-yearly surveillance	1.42%	22,415	18.1	0.2	53.5	22,343	
Non-inferiority based on cost-effectiveness using threshold of €20,000 per QALY gained and base case disutilities (3.1 days lost per colonoscopy)							
5-yearly surveillance	1.0%	22,424	26.3	0.3	54.1	22,343	8826
10-yearly surveillance	1.62%	22,408	18.2	0.2	59.2	22,331	
Non-inferiority based on 80% of base disutilities (2.5 days lost per colonoscopy)							
5-yearly surveillance	1.0%	22,424	21.0	0.3	54.1	22,348	21,206
10-yearly surveillance	1.40%	22,416	14.5	0.2	52.8	22,348	
Non-inferiority based on 120% of base disutilties (3.7 days lost per colonoscopy)							
5-yearly surveillance	1.0%	22,424	31.5	0.3	54.1	22,338	16,754
10-yearly surveillance	1.45%	22,414	21.7	0.2	54.2	22,338	

#### Calculate the non-inferiority margin

To demonstrate non-inferiority of 10-yearly surveillance compared to 5-yearly surveillance in low-risk adenoma patients, a non-inferiority margin of 0.42% (1.42–1%) should be used in the power calculations. Assuming this non-inferiority margin and an expected CRC risk of 1.0% with the standard intervention, 9617 individuals need to be included in each arm of the trial for a total sample size of 19,234 adenoma patients (Table 2).

### Alternative non-inferiority margins

Assuming a lower disutility for being in colonoscopy surveillance (disutility of 2.5 days or 80% of base-case value) to correspond with a larger fraction of the population, QALYs were equal at a lower 10-year CRC risk for the 10-yearly surveillance arm of 1.4% (Table 2). The associated sample size with this non-inferiority margin of 0.4% would be 21,206 adenoma patients. Alternatively, assuming a higher disutility for being in colonoscopy surveillance (disutility of 3.7 days or 120% of base-case value) yields a non-inferiority margin of 0.45% and a corresponding sample size of 16,754 adenoma patients (Table 22).

Basing the non-inferiority margin for EPoS I on cost-benefit with a standard Dutch threshold of €20,000 per life year gained, rather than net benefit alone, would lead to a higher CRC incidence risk of 1.62% allowed in the non-standard arm for non-inferiority (Table 2). With this non-inferiority margin of 0.62%, the required sample size for 90% power would be 8826 adenoma patients (4413 per arm).

### Uncertainty analysis on disutility and colonoscopy sensitivity

**Fig. 3:**
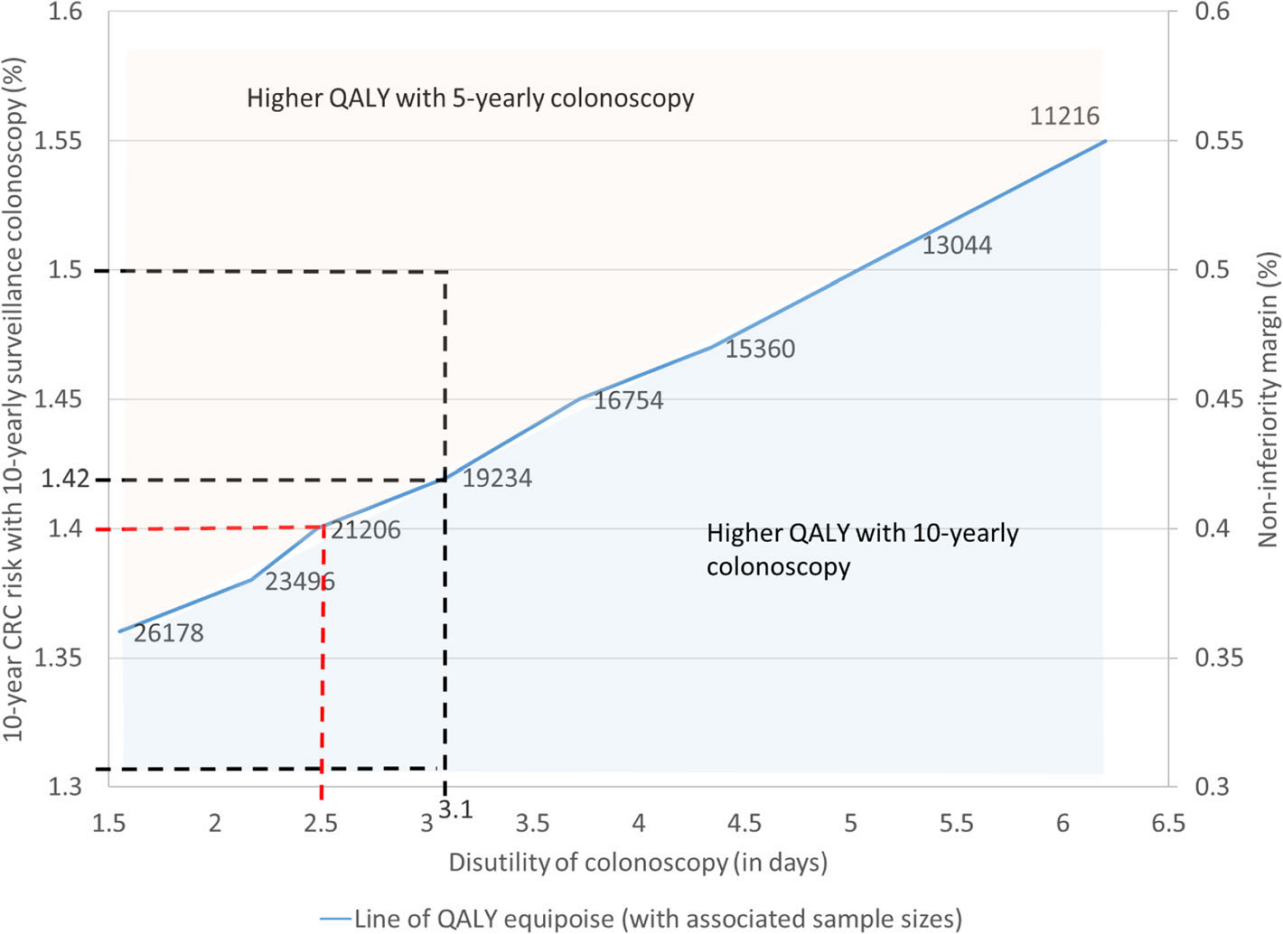
Impact of CRC risk and disutility of colonoscopy on QALYs gained with 5-yearly and 10-yearly colonoscopy surveillance of adenoma patients. Line of QALY equipoise gives for each level of disutility the level of CRC risk in 10-yearly surveillance arm for which QALYs with 10-yearly surveillance are equal to QALYs with 5-yearly surveillance. Values given with this line are associated sample sizes to demonstrate non-inferiority with 90% power. Different dashed lines concern scenarios discussed in Example section of paper

**Fig. 4:**
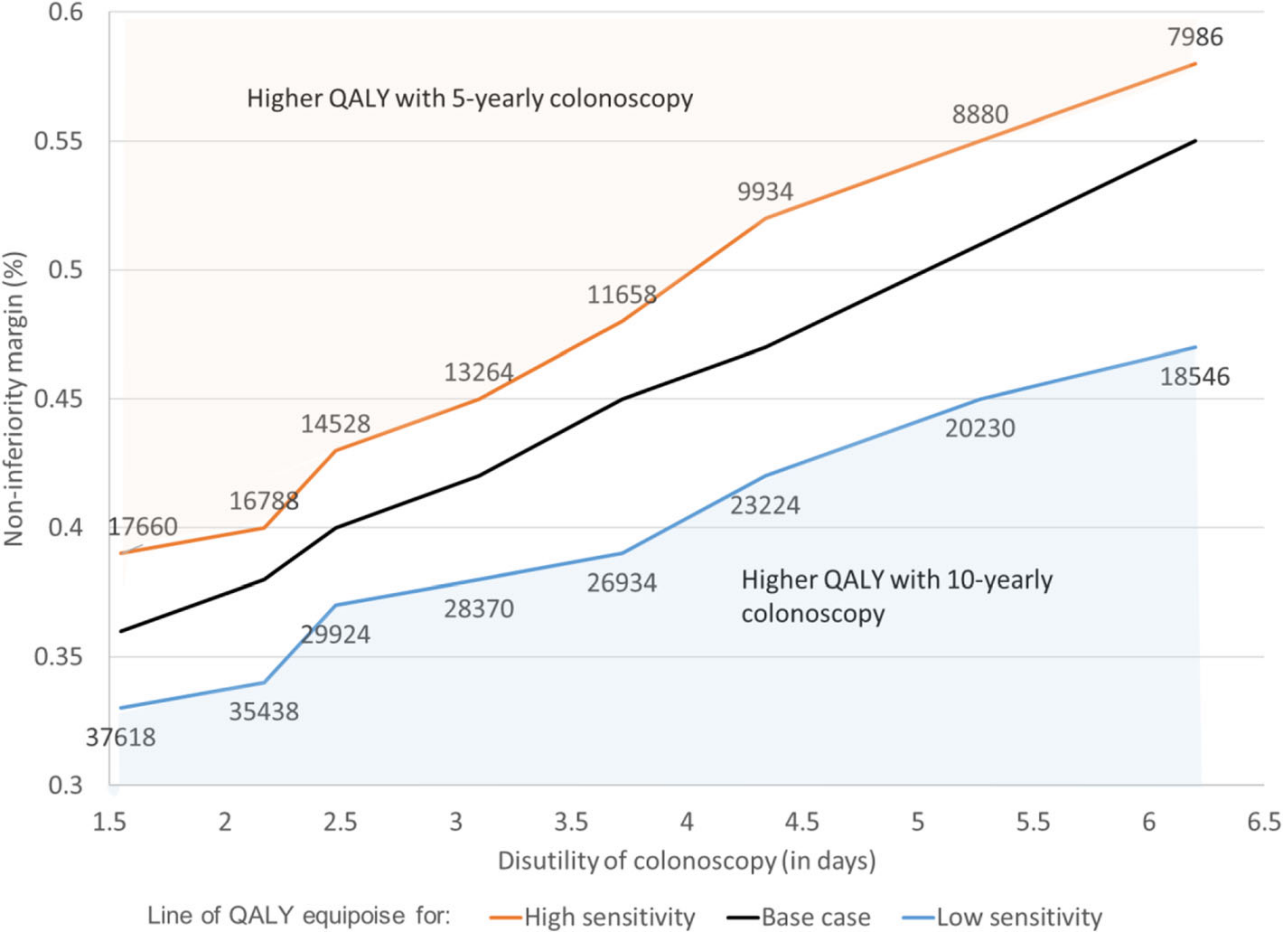
Impact of sensitivity and disutility of colonoscopy on appropriate non-inferiority margin for 10-yearly vs. 5-yearly surveillance. Line of QALY equipoise gives for each level of disutility the level of CRC risk in 10-yearly surveillance arm for which QALYs with 10-yearly surveillance are equal to QALYs with 5-yearly surveillance. Values given with this line are associated sample sizes to demonstrate non-inferiority with 90% power

Figure 3 shows the line of equipoise as a function of the self-perceived disutiities of colonoscopy. The figure shows the translation between disutility of colonoscopy and the resulting QALY gained from 5-yearly surveillance, as well as the associated non-inferiority margin and required sample size to demonstrate non-inferiority. In general, lower assumptions for disutilities associated with colonoscopy resulted in proportionally lower levels of CRC incidence risk allowable in the non-standard arm for equal QALYs for 5- and 10-yearly surveillance. Figure 4 shows the same results in addition to results when assuming 5% higher and 5% lower estimates for colonoscopy sensitivity. Figure 4 clearly demonstrates the sensitivity of required sample sizes for assumptions about colonoscopy sensitivity and disutility One could consider using a sample size of around 30,000 to err on the conservative side in the power calculation in case colonoscopy disutility was lower and sensitivity higher than expected.

## Discussion

This study illustrates the application of a formal method to transform the overall harms and benefits of competing interventions into a measure of the non-inferiority margin. This method offers the benefits of allowing for more explicit and rigorous quantification of various considerations for determining that an intervention is non-inferior. It is applicable to all non-inferiority trials investigating an intervention that is expected slightly less effective as the standard intervention but is associated with less burden and side effects, either because of a de-escalation of the intervention or because of an alternative less invasive treatment regimen. The method may also be used for interventions that are slightly less effective but have (considerably) lower costs.

Our suggested approach for setting the non-inferiority margin is not substantially different from the Delphic method or the approach currently used by trialists who focus on disease outcomes alone. Both aim to set the margin such that the lower effectiveness is compensated by the lower burden, adverse effects, or resource requirements. The important difference between those approaches and the approach in this paper is the explication of all assumptions in our approach, and the consideration of lifetime effects. Using a decision model to estimate QALYs for the standard and alternative interventions ensures that the assumed trade-off between harms and benefits can be reproduced and the enduring impact of differences in interventions on quality of life can be incorporated.

There are several examples of successfully implemented Quality of Life (QoL) clinical trials, where non-inferiority margins are given in QALYs [23–27]. Our approach differs fundamentally from these trials in three ways. First, our approach uses QALYs to actually derive the non-inferiority margin, rather than just defining the non-inferiority margin in terms of QALYs. Second, these studies lack strict standards and a reproducible methodological framework. Finally, and most importantly, these studies suggest the establishment of a non-inferiority margin based on an acceptable loss in QALYs, whereas we suggest that the very definition of a non-inferiority margin requires equipoise in QALYs between the standard and the alternative treatment when the end-points are measured in terms of incidence or mortality outcomes.

This paper suggests an approach for setting a non-inferiority margin for non-inferiority trials, such that appropriate sample size can be estimated in a robust and reproducible way. These considerations for trial design should not be confused with Value of Information analyses which also use decision models [28]. These approaches, such as Expected Value of Sample Information, have been suggested to evaluate whether the resources needed to conduct a trial weigh up against the expected benefits of the additional information to be gained to advance medical decision making.

The impact of our proposed framework on the required sample size for non-inferiority trials depends on the trade-off between lower LY because of lower effectiveness and increase in quality-of-life because of reduced burden of the alternative intervention. In our example, we balance the small QALY gains by forgoing one colonoscopy for everyone versus the large QALY loss for the very few extra people diagnosed with CRC. This resulted in a relatively modest non-inferiority margin and a higher required sample size than currently used in the EPoS trial. However, for other examples of non-inferiority trials where the tradeoffs are much starker, such as a trial for local/regional HPV associated oropharynx cancer [29], the trade-offs may be much more pronounced. Here, the standard therapy is radiotherapy with cisplatin, while the alternative is radiotherapy with cetuximab. The primary end point is overall survival, under the assumption that a cetuximab-based radiotherapy will lead to less morbidity and better quality of life without a significant difference in overall survival or locoregional control. In this case, the increase in quality-of-life from the alternative intervention is very substantial and non-inferiority margin can be considerably wider. Accordingly, this will result in smaller required sample sizes.

In our example, we saw that a large reduction in the necessary sample size of the trial resulted from the incorporation of monetary costs in addition to adverse effects on health-related quality of life as the criterion for determining the non-inferiority margin. Boyd et al. [30] previously demonstrated the use of economic considerations to guide non-inferiority margins. Using cost-utility as an outcome requires defining an acceptable threshold for the cost of a loss in QALYs from the standard to the alternative intervention. Securing consensus on an appropriate cost-per-QALY threshold is fraught with difficulty, especially in the United States. Moreover, empiric data suggest that the threshold cost for forgoing health benefits, such as in the context of a non-inferiority trial, may in fact be higher than the price for gaining health benefits [31, 32]. However, it is important to recognize that medical interventions are not cost-neutral to society nor to patients themselves, who may experience direct financial toxicity from medical expenses, non-medical expenses, co-pays, loss of income, and other mechanisms. Therefore, it may be appropriate to consider whether such costs (at the societal or at least individual patient level) may be relevant to incorporate into clinical trial design.

The EPoS I example with the MISCAN-Colon model illustrates the feasibility of our suggested approach. It does not include establishment of effectiveness compared to a placebo control, as the FDA 95–95 and synthesis methods. However, the issue of ensuring that the non-inferiority margin does not overlap with the relapse rate of a placebo control can be an additional constraint after the size of the margin is determined by weighing the lower benefits of the alternative therapy against its putative lower morbidity burden.

In our case, we used an established sophisticated decision model which was accessible to us. While this model cannot be directly generalized to other situations, our proposed framework can be readily applied to other decision models and diseases. One could develop simpler decision models using Excel, R, or TreeAge, especially for treatment interventions, or use previously developed models and apply these within this framework [19, 33–35]. The most important requirements are case-by-case parameters on effectiveness and disutility of the standard intervention and on disutility of the alternative intervention. In these instances, there is value in trialists and modelers collaborating to formally apply this framework.

While some may argue that disutilities are inherently subjective, difficult to measure, and may have wide variability across the population, they are, by definition, an integral contributor to the size of the non-inferiority margin. The decision analytic framework approach proposed in this paper will allow trial designers to break down and understand the sensitivity of the non-inferiority margin to its component contributors (i.e. the chance of various events occurring and their associated disutilities), rather than using clinical ad hoc judgment to conjecture an estimate of this entire complex quantity at once.

Unfortunately, disutility estimates are often lacking like in our example. However, this framework would still allow postulation of a range of utilities and relate these to the non-inferiority margin and the associated trial sample size. Such an approach would make the implied disutilities of the alternative intervention explicit in the choice for the non-inferiority margin.

In the meantime, inclusion of patient-reported outcome measures that can be expressed and interpreted as a utility in early phase trials can provide pivotal data to help size later trials. However, there are a number of research challenges to be surmounted before population-level utility estimates required for a decision-analytic approach are available to justify sample size in non-inferiority trials. First, disutilities associated with acute effects of cancer screening or treatment have not been well studied, and this is particularly true of serious late treatment effects (e.g. cardiopulmonary toxicity and second malignancies) [36, 37]. Second, point estimates represent the average utility for a population [38, 39], yet equally important is the variability in those utilities across the population and within specific subgroups [40–42], as well as at specific points in the treatment trajectory [43]. Third, although a growing number of health-related quality of life measures can be summarized and interpreted as both a score and a utility [44, 45], most measures of other constructs relevant to gauging the value of a cancer therapy (e.g. cost, treatment burden/complexity) [46, 47] do not yet have established utilities. Work to expand the range of patient-reported outcome measures [48–50] with associated utilities is warranted. Lastly, utilities are often seen as a corollary, rather than an essential component of a non-inferiority trial [51–55].

## Conclusions

In sum, to maximize the rigor and efficiency of future clinical trials seeking to evaluate outcomes after de-escalation of (cancer) interventions, explicit quantification of benefits and harms of an intervention and its omission, along with the impact of each on quality of life, can be a particularly helpful approach. For example, interest has grown in recent years regarding the potential to omit adjuvant radiation therapy after breast conserving surgery for patients with early-stage breast cancer with favorable biologic features [56, 57]. Indeed, it was this particular issue that initially motivated the current exercise—as a means by which to refine the approach towards developing a feasible and sensible trial design in this context. In a trial evaluating such an option, the acceptable increase in breast cancer recurrence risk from omission of therapy can be estimated by weighing the disutility of excess cancer recurrence against the improvements in quality of life from avoidance of the intervention and its side effects. For such evaluations, it is important that trials are not censored at first events but continue to collect information on health outcomes, especially patient-reported quality of life measures and information on other events, after that.

In conclusion, we feel that those considering developing non-inferiority trials could consider innovative approaches to non-inferiority trial design such as the one we outline here, to design research that is simultaneously efficient, rigorous, and meaningful for patients facing complex cancer surveillance, prevention, and management decisions.

## Abbreviations

CISNET: Cancer Intervention and Surveillance Modeling Network
CRC: Colorectal cancer
EPoS study: European Polyp Surveillance study
QALYs: Quality-adjusted life years
QoL: Quality of life
